# Cerebral Lipiodol Embolism in Hepatocellular Carcinoma Patients Treated with Transarterial Embolization/Chemoembolization

**DOI:** 10.1371/journal.pone.0129367

**Published:** 2015-06-24

**Authors:** Hai-Jui Chu, Chung-Wei Lee, Shin-Joe Yeh, Li-Kai Tsai, Sung-Chun Tang, Jiann-Shing Jeng

**Affiliations:** 1 Stroke Center and Department of Neurology, National Taiwan University Hospital, Taipei, Taiwan; 2 Department of Medical Imaging and Radiology, National Taiwan University Hospital, Taipei, Taiwan; Sungkyunkwan University, REPUBLIC OF KOREA

## Abstract

**Background and Purpose:**

Liver cancer is the third leading cause of cancer mortality worldwide. The aim of this study was to investigate the frequency and characteristics of cerebral lipiodol embolism (CLE) in patients with hepatocellular carcinoma (HCC) receiving transarterial embolization/chemoembolization (TAE/TACE).

**Methods:**

We reviewed all HCC patients who received TAE/TACE during the period of 2007 and 2013 at a university medical center. The frequency of CLE per procedure and the clinical manifestations of CLE, including the review of previous reported cases (n = 24), were analyzed.

**Results:**

During the study period, a total of 7855 TAE/TACE procedures were conducted on 3277 patients. There were 8 patients (mean age 59±11 years; 5 males and 3 females) who developed CLE. The frequency of TAE/TACE-related CLE was 1.02 (95% CI, 0.44–2.01) per 1000 procedures. Acute disturbance of consciousness and respiratory distress after TAE/TACE were the most common presentations of CLE. All patients had disseminated infarcts involving both the anterior and posterior cerebral circulations. For 3 patients with shunting between the tumor feeding artery and the pulmonary vein, a specific imaging pattern of coexisting scattered hyperdense spots was found. Furthermore, combined with our 8 cases, the total of 32 cases indicated that old age and female sex were the two risk factors for poor outcome after CLE.

**Conclusions:**

CLE is a rare but potentially serious complication in HCC patients receiving TAE/TACE. The clinical characteristics of CLE summarized in our study would help facilitate the ability of clinicians to provide timely diagnosis and management.

## Introduction

Liver cancer is the third leading cause of cancer mortality worldwide [1] and is ranked as the second highest annual increase rate cancer [2]. Hepatocellular carcinoma (HCC) attributes to the 70% to 85% major subtype of primary liver cancers worldwide [3]. Information about the disease, treatment, and the related complications has progressively become a critical issue. Transarterial embolization (TAE) or transarterial chemoembolization (TACE) is the current mainstay treatment for HCC when surgery or tumor ablation is not suitable. This procedure consists of the injection of lipiodol, an iodized oil mixed with or without chemotherapeutic agents, into the tumor feeding artery. It leads to embolization of the blood supply of the tumor and induces a cytotoxic effect and tumor regression [4, 5].

Although rarely reported, this procedure may be complicated by cerebral lipiodol embolism (CLE), which refers to embolic materials entering the circulatory system and occluding the cerebral arteries. In the past decade, there have been only several case reports describing the clinical presentation of CLE after the TAE/TACE procedure for HCC [6–23]. The frequency of CLE after TAE/TACE has not been established, although HCC patients requiring this treatment are numerous. In the present study, we reviewed all HCC patients undergoing TAE/TACE within a 7-year period at a university medical center and investigated the frequency and clinical manifestations of patients suffering from CLE. In addition, we identified a particular head imaging pattern in a subgroup of CLE patients, which was specifically linked to the direct HCC invasion of the diaphragm and pleura.

## Materials and Methods

### Ethics Statement

The informed consent was not obtained because patients’ information was anonymized and de-identified prior to analysis. This study was approved by the Institutional Ethics Committee of National Taiwan University Hospital.

### Patients

We retrospectively reviewed all HCC patients who received the TAE/TACE procedure during the period of January 2007 to July 2013 at National Taiwan University Hospital. Patients who had acute neurological symptoms and underwent head imaging examination (computed tomography [CT] or magnetic resonance imaging [MRI]) at the same admission were carefully reviewed by two of the authors, HJC (neurologist) and CWL (neuroradiologist) to confirm the diagnosis of CLE.

### Diagnosis of CLE

The diagnosis of CLE was based on the head imaging findings. According to previous studies [6, 20], CLE is characterized by disseminated lesions across multiple vascular territories, mainly at the distal gray matter. Lipiodol deposition is shown as hyperintensity on head CT images. On head MRI scans, we sought evidence of acute cerebral ischemic change which would have decreased water diffusion in infarcted tissue, increased signal in diffusion-weighted imaging (DWI), and decreased apparent diffusion coefficient (ADC) signals. Comparison of these images with signals on T2 and T2 fluid attenuation inversion recovery (FLAIR) images was also performed. With clinical history, CLE diagnose was made. Other cerebral insults such as intracranial metastasis or different types of stroke were excluded. Intracranial metastasis is often presented as single or multiple iso- or hypodense mass at grey-white matter interface with significant surrounding edema on non-contrast CT and high signal intensity with marked hyperintense at adjacent areas on T2 weighted image (WI) of MRI. Both head CT and MRI showed strong contrast enhancement. For other types of ischemic stroke, there is usually one lesion in a single arterial territory, though some may present as few but not disseminated lesions involving more than one vascular territory such as cardioembolic stroke or borderzone infarcts.

### TAE/TACE procedure

The tumor supplying artery was approached with a 4-Fr J-curve (5Fr RLG) and 2.7 Fr microcatheter (Progreat; Terumo, Japan). The TAE/TACE procedure was performed by infusion of iodized oil contrast medium (Lipiodol Ultra-Fluide; Guerbet, Aulnay-sous-Bois, France) and/or doxorubicin (Adrinamycin) followed with gelatin sponge particles. The amount of lipiodol injection ranged from 10–90 ml and some patients also received doxorubicin 40 mg. Tumor angiography was reviewed and extra-hepatic shunting was specially recorded.

### Data Acquisition

Demographic data, including stroke risk factors and HCC status, were recorded. Patients’ baseline daily activities were reviewed by medical charts and were measured as Performance Status (PS) which is based on the Eastern Cooperative Oncology Group score [24]. Tumor staging of HCC was defined by Barcelona Clinic Liver Cancer (BCLC) stage [25]. In addition, embolized tumor location, vascular supply, and whether lung pleura invasion/pulmonary vein shunting was performed were recorded. The head and chest images were evaluated by a specialist (CWL) to analyze the intracerebral lipiodol embolic distribution as well as additional high signal spots mainly at the cerebral hemispheres. Evidence of pneumonitis was examined. Post-CLE outcomes were determined as 3-month fatality in our cases. Whether patients died or were in a vegetative state at the end of the description period (about 6 weeks after the onset of CLE) was recorded as the outcome.

### Literature Review

A literature review was performed via Medline and PubMed. The following word combinations were searched: “cerebral lipiodol embolism”, “cerebrovascular accident”, “intracranial embolism”, “hepatocellular carcinoma”, “therapeutic chemoembolization”, “lipiodol”, “ethiodized oil”. The bibliographies of reviewed articles were also explored.

Combined reported cases with ours, the clinical characteristics and factors related to the outcome of all patients were analyzed.

### Statistical analysis

Categorical variables are presented as percentage, and continuous and discrete variables as mean ± standard deviation. The frequency of CLE in patients with HCC was determined by dividing the number of strokes by the number of TAE/TACE procedures. We analyzed the frequency and its 95% confidence interval (CI) of CLE in HCC patients. Factors related to outcome among all cases were also analyzed using t test as well as chi-square test (Fisher's exact test) for categorical variables. A *P*-value of less than 0.05 was considered to indicate a significant difference statistically. MedCalc Statistical Software version 13.1.2 (MedCalc Software bvba, Ostend, Belgium; http://www.medcalc.org; 2014) was used for analysis.

## Results

### Frequency of CLE in HCC Patients after TAE/TACE

During the study period, a total of 7855 TAE/TACE procedures were performed in 3277 patients (mean age: 64.2±12.3 years; 71.5% males). There were 8 patients (mean age: 58.6±11.8 years; 5 males and 3 females) who developed CLE after the procedure. The frequency of CLE (per 1000 procedures) after TAE/TACE was 1.02 (95% CI, 0.44–2.01) for all patients, 1.36 (95% CI, 0.28–3.97) for females, and 0.89 (95% CI, 0.29–2.07) for males.

### Clinical Characteristics and Outcome in CLE patients

In 7855 procedures of TAE/TACE, there were 30 cases receiving head CT or MRI after TAE/TACE due to acute neurological symptoms. Among them, there were 22 (73.0%) with disturbance of consciousness, 6 (20%) with hemiparetic weakness, and 2 (6.7%) with headache. Among those 30 cases, there were 13 (43.3%) with positive findings on either CT or MRI, including 8 CLE, 3 intracranial hemorrhage, and 2 intracranial metastasis.

Table 1 lists the clinical manifestations of the 8 CLE patients. All patients were able to perform independent daily activities prior to CLE, and only 1 patient had conventional stroke risk factors. Most patients (75%) had stroke symptoms within 6 hours after TAE/TACE. Acute disturbance of consciousness occurred in all patients. In contrast with conventional stroke, hemiparesis was not a common presentation (2 patients, 25%). Other neurological symptoms which usually described were aphasia (75%), disorientation (62.5%) and visual disturbance (37.5%). Besides, respiratory symptoms or signs were mentioned by the 6 (75%) patients during or after TAE/TACE.

**Table 1:** Clinical and Radiological Manifestation of Eight Patients with Cerebral Lipiodol Embolism.

Patient	1	2	3	4	5	6	7	8
**Age (y)/Sex**	39/M	51/M	73/F	67/F	54/F	63/M	52/M	72/M
**Stroke risk factors**	No	No	No	No	No	Yes^a^	No	No
**Liver Cirrhosis**								
Etiology	HBV	HBV	HCV	HCV	HBV	HBV	HBV	HBV
Child-Pugh classification	A	A	A	B	A	A	A	B
**HCC status**								
Tumor description^b^	Multiple	Multiple	Multiple	Multiple	Multiple	Single	Multiple	Multiple
PV invasion/thrombosis	Yes/No	Yes/Yes	No/No	No/No	No/No	No/No	No/No	No/No
ECOG PS	1	1	0	2	2	1	0	0
BCLC staging	C	C	B	C	C	C	B	B
Received procedure	TAE	TACE	TACE	TACE	TACE	TACE	TACE	TACE
Size of embolized HCC (cm)	8	13	19	6	3	14	17	10
***Shunt to pulmonary vein***	Yes	Yes	Yes	No	No	No	No	No
Number of TAE/TACE	7	4	4	11	4	3	2	2
Delivering vessel	RIPA	RHA,LHA	RIPA	LGA	LIPA	RSGA	RHA	RHA
Dose of lipiodol (ml)^c^	13	30	15	10	90	50	30	20
**Stroke manifestation**								
Onset within 6 h after TACE	Yes	Yes	Yes	Yes	Yes	No(25h)	No(144h)	Yes
Coma scale while CLE	E4M4V3	E3M5V4	E3M6V4	E3M6V5	E4M5V3	E4M5V4	E4M6V4	E4M6V4
Visual disturbance	Yes	Yes	No	No	No	No	Yes	No
Hemiparetic weakness	No	Yes	No	Yes	No	No	No	No
Respiratory distress while TACE	Yes	Yes	Yes	No	Yes	No	No	Yes
**Brain image finding**								
Hyperdense spots	Yes	Yes	Yes	No	No	No	No	No
**Chest image finding**								
Lipiodol pneumonitis^d^	N/A	Yes	Yes	Yes	N/A	Yes	Yes	Yes
**Outcome**								
3-month fatality	No	No	No	No	No	No	No	No
Death or vegetative status^e^	No	No	No	No	Yes	No	No	Yes

M, male; F, female; HCC, hepatocellular carcinoma; PV, portal vein; BCLC, Barcelona Clinic Liver Cancer; ECOG PS: Eastern Cooperative Oncology Group Performance Status; TAE: trans-arterial embolization; TACE: trans-arterial chemoembolization;

RIPA: right inferior phrenic artery; LIPA: left inferior phrenic artery; RHA: right hepatic artery; LHA: left hepatic artery; LGA: left gastric artery; RSGA: Right superior gluteal artery

^a^ Patient No 6 had hypertension, diabetes mellitus, congestive heart failure and dyslipidemia

^b^ Multiple nodules defined as ≥ 3

^c^ TACE was referred as lipiodol mixed with doxorubicin 40 mg followed with injection of Gelfoam particles

^d^ Pneumonitis was diagnosed by chest CT and plain film after the procedure

^e^ The time determined the outcome was at discharge

**Fig 1:**
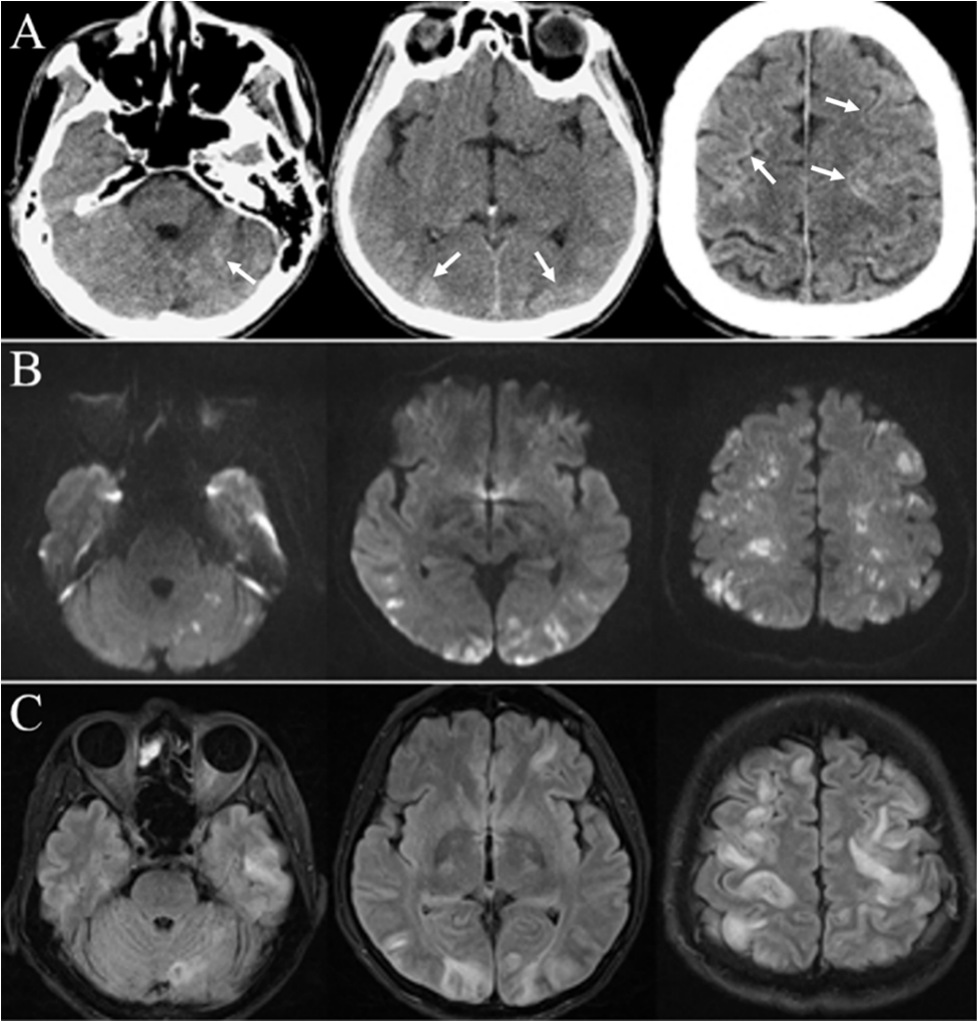
Head imaging findings in a patient with hepatocellular carcinoma who had cerebral lipiodol embolism (CLE) after transarterial chemoemobolization. Head CT and MRI were performed at 9 hours and 1 day after the symptoms, respectively. Row A: Head non-contrast CT showed disseminated high-density lesions mainly at gray matter (arrow), Row B and C: diffusion weighted imaging (DWI) and T2 fluid attenuation inversion recovery (FLAIR) of head MRI. The scans showed multiple disseminated hyperintensity/high signal lesions mainly at the gray matter of the cerebrum and cerebellum. The larger areas on FLAIR than on DWI for the same lesions indicate the existence of peri-stroke edema.

**Fig 2:**
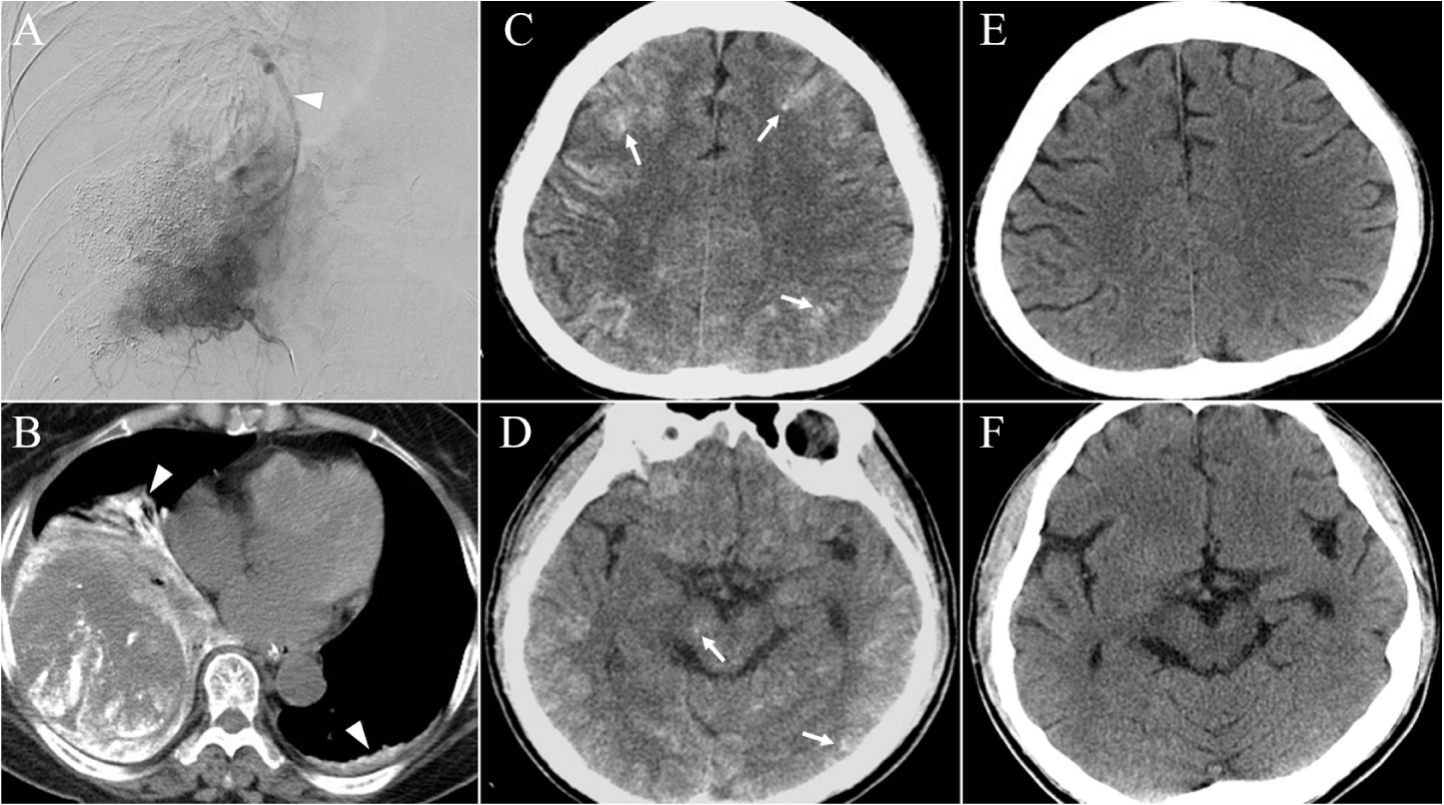
Head and abdominal imaging findings in HCC patients with pulmonary vein shunting due to direct diaphragm and pleura invasion. A right inferior phrenic artery angiogram showed prior embolized hepatic tumor with large adjacent recurrence. An early opacified pulmonary vein branch was seen (arrow head) (A); non-contrast chest CT at 2 days after CLE showed lipiodol pneumonitis at bilateral collapsed basal lung (arrow head) (B); on head non-contrast CT there were several hyperdense spots (arrow) in addition to typical disseminated lesions of increased attenuation of CLE at the cerebral hemispheres (C) and brain stem (D). At 3 weeks follow-up, head CT of the same patient showed disappearance of the previous hyperdense lesions (E & F).

Regarding the imaging findings, all patients had disseminated high-density lesions mainly at gray matter involving the cerebral hemispheres, cerebellum, brainstem, and thalamus on CT with fluid restriction on DWI in MRI (Fig 1A and 1B). Bigger areas of hyperintensity on T2 sequence than those on DWI sequence indicating the edematous change after CLE were also noted (Fig 1C). Importantly, we identified a particular imaging pattern that was characterized by scattered hyperdense spotted brain lesions with extremely high signal intensity in 3 patients who had pulmonary vein shunting due to direct diaphragm and pleura invasion (Fig 2). However, no obvious difference was found in clinical parameters between patients with or without these specific imaging findings.

There were 3 (37.5%) out of the 8 patients having follow up head image. Two had non-contrast CT in 12, and 17 days post CLE, respectively and all lesions were resolved completely. The other one received head MRI in 17 days post CLE, and it still showed faint restriction on DWI and with mild swelling along the cortex.

There was no case fatality within 90 days. Six patients regained their pre-CLE consciousness status within 3 weeks. Two patients had poor functional outcome at discharge. One continued to have disturbance of consciousness with comorbid systemic infectious disease and respiratory failure. The other remained bedridden and required external oxygen support.

### Literature Review and Factors Associated with Poor Outcome

Finally, we reviewed all CLE cases reported previously and summarized the clinical parameters in Table 2. There were 18 articles with a total of 24 cases of CLE after TAE/TACE that were published from 2004 to 2014 [6–23]. Our case series showed high concordance with the clinical characteristics, presentations, and outcomes from the 24 previously reported CLE cases. Furthermore, a total of 6 (18.8%) CLE patients were dead or in a vegetative state at the end of the description. Factors of old age (68.8±8.8 versus 58.5±10.8 years, P = 0.038) and female sex (83.3 versus 23.1%, p = 0.011) but not other clinical parameters were significantly associated with poor outcome based on univariate analyses.

**Table 2:** Clinical Manifestations of 32 Reported Cases with Cerebral Lipiodol Embolism.

	Present study cases (n = 8)	Previous reported cases(n = 24)	Total cases (n = 32)
Age	58.6±11.8	60.9 ±11.1	60.4±11.1
Male	5 (62.5%)	16/24 (66.7%)	21/32 (65.6%)
Course of TAE/TACE≧3 times	6 (75.0%)	13/23 (56.5%)	19/31 (61.3%)
Dose of lipidol≧20ml	5 (62.5%)	13/19 (68.4%)	18/27 (66.7%)
Embolized tumor location at right lobe	5 (62.5%)	14/16 (87.5%)	19/24 (79.2%)
CLE onset time≦6 hours	6 (75.0%)	22/24 (91.7%)	28/32 (87.5%)
Respiratory symptoms and/or pneumonitis	8 (100%)	13/15 (86.7%)	21/23 (91.3%)
Consciousness change	8 (100%)	18/24 (75.0%)	26/32 (81.3%)
Hemiparetic weakness	2 (25.0%)	7/24 (29.2%)	9/32 (28.1%)
Poor outcome^a^	1 (12.5%)	5/24 (20.8%)	6/32 (18.8%)

CLE: cerebral lipiodol embolism; TAE/TACE: transarterial (chemo)embolization.

^a^Poor outcome indicated death or vegetative status at the end of the case description

## Discussion

Lipiodol is a mixture that contains 37% iodine (475 mg/ml) and ethyl esters of the fatty acids of poppyseed oil. It had been widely used for contrast medium of lymphangiography until late in the 20th century [26]. There was an important safety issue: extravasation of lipiodol from lymph into the venous system may cause lipiodol embolism involving brain, lung, and kidney [26–28]. CLE related to lymphangiography had been reported for 30 cases but is not mentioned anymore because of technical improvement and the accessibility of non-invasive CT and MRI imaging [26].

The TAE method has been applied in non-operative HCC since the year 1979 and the method of TACE, which used lipiodol mixed with chemotherapy agents, was introduced in the year 1983 [29, 30]. These reports described the clinical presentation of CLE and also included the pathological profile to confirm the longstanding statement that lipiodol is the component occluding the cerebral artery and leading to the unique feature on imaging. However, all of the previous studies only described a single case or few cases and the frequency of this complication in HCC patients receiving TAE/TACE has not been previously investigated.

In our study, we not only reported 8 new cases of CLE, but also showed that the frequency of CLE after TAE/TACE was 1.02 (95% CI, 0.44–2.01) per 1000 procedures. Interestingly, this number was similar to that found in a previous study on pulmonary lipiodol embolism; in 2300 procedures of TACE on 850 patients with liver tumor, the frequency was found to be 1.74 per 1000 procedures [29].

The route of lipiodol through tumor embolized artery to brain has been widely discussed. Previous reports stated that the existence of shunting between systemic vessels and pulmonary vessels would facilitate the extravasation of lipiodol into the systemic circulation and increase the risk of CLE [9, 21]. In our case series, 3 patients had tumor invasion of right lung, and angiography revealed the tumor-feeding artery directly shunting to the pulmonary vein. Importantly, we also identified a particular imaging pattern on head CT of scattered hyperdense spots in these 3 patients. This finding provides supporting evidence that tumor-feeding artery shunting to the pulmonary vein may lead to a larger amount of lipiodol deposition in brain during TAE/TACE procedures. However, the clinical impact of this particular imaging pattern was not obvious in our study patients.

Regarding the aspect of clinical symptoms, acute disturbance of consciousness and respiratory distress within a very short time period after the TAE/TACE procedure are the most common presentations of CLE. Interestingly, with the combination of previous reported cases, there were 4 out of the 32 CLE cases (12.5%) having neurological symptoms after 24 hours of TAE/TACE. Out of the 4 cases, 3 described respiratory symptoms at 25, 35 and 48 hours respectively prior to the development of CLE symptoms, which indicated the possibility of stepwise lipiodol washing out from liver, lung and to brain consequently [14]. Besides, since the symptoms of CLE were often subtle or atypical from the conventional stroke syndrome, delayed recognition of the occurrence of CLE may also be possible. Therefore, clinicians should be aware of the characteristic presentation and time course of CLE in HCC patients receiving TAE/TACE for early detection of this rare complication. For patients with clinically suspected CLE, head imaging should be performed without any delay.

Considering the findings on head imaging, diagnosis of CLE could be easily established from its unique pattern of diffuse hyperdensity lesions on non-contrast head CT. In the few cases in which patients undergo only head MRI, CLE needs to be differentiated from several etiologies such as hypoperfusion (watershed)-related ischemia, cerebral embolism including cerebral fat embolism, and posterior reversible encephalopathy syndrome [6]. However, with a patient's history, clinical data, and imaging findings, it should not be a difficult task to distinguish CLE from other conditions. Moreover, it is reasonable that the imaging findings of disseminated lesions reflect the typical symptom of disturbance of consciousness rather than focal neurological deficit in patients with CLE.

Combined with previously reported CLE cases, CLE patients are usually aged around 60 years, male, have multiple TAE/TACE procedures, and receive right liver lobe procedure with a dose of lipiodol over 20 ml. In addition, older age and female were associated with poor outcome in CLE patients. No other parameters related to TAE/TACE procedures or clinical manifestations of CLE were associated with outcome. Further study with prospective study design and multicenter case recruitment should be performed to clarify which HCC patients have a high risk of developing CLE after TAE/TACE and identify useful outcome predictors of CLE. Treatment experience with regard to CLE in the literature has mostly supported such therapies as hydration, oxygenation, intubation, and sedation as needed [9, 13–15, 17, 18, 20–23]. Osmotic therapy, neurotrophic agents, and vascular dilators have also been mentioned without clear evidence of their effect.

There are several limitations of our study. First, CLE patients were identified only when they had clinical symptoms and positive imaging findings, therefore, patients with minor or no symptoms would have been missed. Due to this factor, the overall frequency may be underestimated. Besides, the frequency of neurologic consult to overall CLE incidence with neurologic symptoms was not known. Secondly, the diagnoses were made by clinical speculation without pathological evidence. Third, our study was performed at a single hospital using a retrospective study design; as we mentioned earlier, further studies with a multicenter design and recruiting more CLE patients may help to validate our observations.

In summary, ours is the first study reporting the frequency of CLE in HCC patients receiving TAE/TACE, and it revealed a particular head imaging pattern in CLE patients with direct diaphragm and pleura invasion. CLE is a rare but potentially serious complication in HCC patients who receive TAE/TACE. The clinical characteristics and prognostic factors from our study can help clinicians achieve timely diagnosis and management of CLE.
